# Genetics and pathologic landscape of lineage switch of acute leukemia during therapy

**DOI:** 10.1038/s41408-024-00983-2

**Published:** 2024-01-25

**Authors:** Ting Zhou, Choladda V. Curry, Mahsa Khanlari, Min Shi, Wei Cui, Deniz Peker, Weina Chen, Endi Wang, Juehua Gao, Qi Shen, Wei Xie, Fatima Z. Jelloul, Rebecca L. King, Ji Yuan, Xiaoqiong Wang, Chen Zhao, Ifeyinwa E. Obiorah, Elizabeth L. Courville, Eric Nomura, Sindhu Cherian, Mina L. Xu, W. Richard Burack, Hong-xing Liu, Elias J. Jabbour, Koichi Takahashi, Wei Wang, Sa A. Wang, Joseph D. Khoury, L. Jeffrey Medeiros, Shimin Hu

**Affiliations:** 1 Department of Hematopathology, The University of Texas MD Anderson Cancer Center, Houston, TX USA; 2 Department of Pathology & Immunology, Baylor College of Medicine and Texas Children’s Hospital, Houston, TX USA; 3 Department of Pathology, St. Jude Children’s Research Hospital, Memphis, TN USA; 4 Department of Laboratory Medicine and Pathology, Mayo Clinic, Rochester, MN USA; 5 Department of Pathology, The University of Kansas Medical Center, Kansas City, KS USA; 6 Department of Pathology and Laboratory Medicine, Emory University, Atlanta, GA USA; 7 Department of Pathology, The University of Texas Southwestern Medical Center, Dallas, TX USA; 8 Department of Pathology, Duke University Medical Center, Durham, NC USA; 9 Department of Pathology, Northwestern University Feinberg School of Medicine, Chicago, IL USA; 10 Department of Pathology, AdventHealth-Orlando, Orlando, FL USA; 11 Department of Pathology and Laboratory Medicine, Oregon Health & Science University, Portland, OR USA; 12 Department of Pathology, Case Western Reserve University School of Medicine, Cleveland, OH USA; 13 Department of Pathology, University of Virginia School of Medicine, Charlottesville, VA USA; 14 Department of Laboratory Medicine & Pathology, University of Washington Medical Center, Seattle, WA USA; 15 Department of Pathology, Yale University School of Medicine, New Haven, CT USA; 16 Department of Pathology and Laboratory Medicine, University of Rochester Medical Center, Rochester, NY USA; 17 Molecular Medicine Center, Beijing Lu Daopei lnstitute of Hematology, Beijing, China; 18 Department of Leukemia, The University of Texas MD Anderson Cancer Center, Houston, TX USA; 19 Department of Pathology, Microbiology, and Immunology, University of Nebraska Medical Center, Omaha, NE USA; 20 Present Address: Department of Pathology and Laboratory Medicine, Memorial Sloan Kettering Cancer Center, New York, NY USA

**Keywords:** Cancer genetics, Acute myeloid leukaemia

Dear Editor,

Lineage switch in leukemia, characterized by a complete cell-fate conversion from one lineage to another, is associated with a dismal prognosis. The era of immunotherapy has witnessed a notable increase in its incidence, approaching 8% of B-ALL following anti-CD19 chimeric antigen receptor T-cell (CAR-T) therapy [1]. This significant challenge underscores the pressing need for a deeper understanding of lineage switch. The existing literature, primarily comprising case reports and small case series, fails to offer a comprehensive portrayal of the clinicopathological features of this phenomenon [1–5]. Furthermore, data on its genetic and molecular basis are scant, with the understanding largely limited to its association with *KMT2A* fusions and *BCR::ABL1* [6, 7]. Thus, we undertook a multi-institutional investigation into lineage switch in acute leukemias, with a two-fold aim: primarily, to achieve an in-depth understanding of its clinicopathologic features, a crucial step for identifying at-risk patients and shaping prevention and management strategies in susceptible populations; and secondly, to identify potential genetic drivers and gain insights into clonal evolution pathways of these leukemias, ultimately paving the way toward targeted therapies.

This study included 33 cases of acute leukemia, which underwent lineage switch, diagnosed from 2003 to 2022. To exclude cases where the second leukemia might be therapy-related, inclusion was limited to patients demonstrating cytogenetic or molecular evidence for clonal-relatedness between their first and second leukemias, as determined by karyotype, fluorescence in-situ hybridization (FISH), and next-generation sequencing (NGS). In two cases (#6 and 18), the clonal relatedness was further substantiated by identical *IGH* gene rearrangements. Furthermore, patients diagnosed with mixed-phenotype acute leukemia for the first acute leukemia, whether bilineal or biphenotypic, were excluded. However, we included cases of bilineal leukemia diagnosed as the second leukemia, provided one population represented residual disease of the first leukemia.

The cohort comprised 22 males and 11 females, with a median age of 34.6 years (range, 0.1–83.2). The first leukemia underwent a lineage switch after a median interval of 7.8 months (range, 0.9–38.2). At the switch point, 79% (26/33) of patients were in complete remission (CR) of the first leukemia, as confirmed by flow cytometry. The switch involved a conversion from ALL to acute myeloid leukemia (AML) in 28 patients (25 with B-ALL and 3 with T-ALL), from lymphoid blast phase (LyBP) to myeloid blast phase (MyBP) of CML in one, and from AML (including one case of myeloid sarcoma) to B-ALL in four (Table 1). Children appeared more likely to present with T-ALL or AML as an initial diagnosis compared to adults and experienced a modestly longer duration between the initial diagnosis and lineage switch (Table S1). Prior to lineage switch, all 33 patients received chemotherapy and six underwent allogeneic hematopoietic stem cell transplantation (HSCT). In addition, 15 of the B-ALL patients also received targeted immunotherapies, including anti-CD19 monoclonal antibodies in ten patients, anti-CD20 in five, and anti-CD22 in five, as well as CAR-T therapy in two (Table 1 and Table S2). In these scenarios, lineage switch is believed to be driven by the immunologic pressure exerted by the targeted therapy, where a phenotypic switch may allow immune escape of leukemic cells. The release of inflammatory cytokine is also thought to contribute to the process [8].

**Table 1:** Clinicopathologic features of patients with lineage-switching leukemia.

Case #	Sex	Age at acute #1	Dx of acute #1	Tx of acute #1	Dz status of acute #1 at dx of acute #2	Dx of acute #2	Interval from acute #1 to #2	Tx of acute #2	Pt status at last F/U	Dz status of acute #1 at last F/U	Dz status of acute #2 at last F/U	OS from acute #1	OS from acute #2
1	M	0.1	B-ALL	Chemo	CR by FC	AML, Mono	7.8	Chemo	DOD#2	CR by FC	Persistent AML	9.1	1.3
2	M	0.1	B-ALL	Chemo, Blina	CR by FC	AML, Mono	3.9	Chemo	DOD#2	MRD+ by FC	Persistent AML	11.0	7.1
3	M	0.2	B-ALL	Chemo	12% by FC	AML, Mono	5.1	Chemo	DOD#1	Switch back	Residual AML	7.9	2.8
4	F	12.8	B-ALL	Chemo, CART	CR by FC	AML, M1	15.5	Chemo	Alive	CR by FC	Persistent AML	17.2	1.8
5	F	27.5	B-ALL	Chemo, SCT	CR by FC	AML, Mono	9.0	Chemo	DOD#2	CR by FC	MS	14.3	5.3
6	M	34.6	B-ALL	Chemo, R, Blina, Ino, SCT	CR by FC	AML, MS, Mono	7.4	Chemo	DOD#2	CR by FC	Persistent AML	10.0	2.6
7	M	40.2	B-ALL	Chemo, Blina	CR by FC	AML, M4	4.3	Chemo	DOD#2	CR by FC	Persistent AML	6.8	2.6
8	F	43.2	B-ALL	Chemo, R, Ino, Blina	CR by FC	AML, Mono	10.1	Chemo	DOD#2	CR by FC	Persistent AML	12.0	1.9
9	M	44.6	B-ALL	Chemo, Blina	CR by FC	AML, Mono	4.6	Chemo, SCT	DOD#2	CR by FC	Relapsed AML	10.6	6.1
10	F	46.2	B-ALL	Chemo, SCT, Blina	4% by FC	MS, Mono	12.4	Chemo	DOD#2	CR by FC	MS	13.4	1.0
11	F	61.8	B-ALL	Chemo, Blina, Ino	CR by FC	AML, M4	6.2	Chemo	DOD#1	Switch back	CR by FC	12.4	6.1
12	F	60.1	B-ALL	Chemo	CR by FC	AML, Mono	8.0	No	DOD#2	MRD+ by FC	Persistent AML	9.3	1.3
13	F	65.9	B-ALL	Chemo	CR by morphology	AML, Mono	0.9	Chemo, SCT	Alive	CR by FC	CR by Fc	23.9	23.0
14	M	1.1	B-ALL	Chemo	CR by FC	AML, Mono	14.7	Chemo, SCT	Alive	CR by FC	CR by FC	43.7	29.0
15	M	0.3	B-ALL	Chemo, CART	CR by FC	AML, Mono	8.5	Chemo, SCT	Alive	CR by FC	CR by FC	15.6	7.1
16	M	7.1	B-ALL	Chemo	CR by FC	AML, Mono	38.2	Chemo	DOD#2	CR by FC	Persistent AML	39.9	1.7
17	F	67.3	B-ALL	Chemo, R, Ino, Blina	0.4% by FC	AML, M0	6.2	Chemo	DOD#2	MRD+ by FC	Persistent AML	10.7	5.0
18	M	15	B-ALL	Chemo, Ino	CR by FC	AML, M4	4.8	Chemo	Alive	CR by FC	CR by FC	6.9	2.1
19	M	36.1	B-ALL^a^	Chemo, R	CR by FC	AML, M4	5.6	Chemo, SCT	DOD#2	CR by FC	Relapsed AML, MS	28.7	23.1
20	M	23.4	B-ALL^b^	Chemo, SCT	CR by FC	AML, Mono	7.7	Chemo	DOD#1	Switch back	Residual AML	9.3	1.7
21	M	69.6	B-ALL^b^	Chemo	1% by FC	AML, M6	12.2	No	DOD#2	MRD+ by FC	AML, not treated	12.4	0.2
22	M	21.2	B-AL L^c^	Chemo, SCT	CR by FC	AML, M2 (MRC)	12.0	Chemo	Alive	CR by FC	Persistent AML	17.4	5.5
23	F	83.1	B-ALL^c^	Chemo, R, Blina	0.2% by FC	AML, M0	4.9	Chemo	DOD#2	MRD+ by FC	Persistent AML	5.9	1.0
24	M	83.2	B-ALL^d^	Chemo, Ofa	CR by FC	AML, M2	7.3	No	DOD#2	CR by FC	AML, not treated	8.0	0.7
25	M	69.1	B-ALL^e^	Chemo, Blina	CR by FC	AML, M6	6.0	NA	DOD#2	CR by FC	Persistent AML	7.2	1.1
26	M	52.5	B-LyBP^f^	Chemo	CR by FC	MyBP	6.2	Chemo	DOD#2	CR by FC	Persistent MyBP	7.7	1.5
27	M	12.8	T-ALL	Chemo	CR by FC	AML, M0	24.3	Chemo, SCT	Dead	NA	NA	117.9	93.7
28	M	13	T-ALL	Chemo	CR by FC	AML, M4	6.0	Chemo	DOD#2	CR by FC	Persistent AML	8.0	2.0
29	F	9.7	T-ALL	Chemo, SCT	CR by FC	AML, M0	14.6	NA	Alive #2	CR by FC	Persistent AML	15.3	0.7
30	F	0.6	AML, Mono	Chemo	10% by FC	B-ALL	9.8	Chemo, SCT	Dead from SCT	CR by FC	CR by FC	18.4	8.6
31	M	1.5	AML, Mono	Chemo	CR by FC	B-ALL	12.9	Chemo, SCT	Alive	CR by FC	CR by FC	179.4	166.6
32	M	73.6	AML, M2	Chemo	CR by FC	B-ALL	13.8	Chemo	Dead	NA	NA	18.3	4.5
33	M	68.1	AML, M0, (MRC)^c^	Chemo	CR by FC	B-ALL	25.8	Chemo	DOD#2	CR by FC	Persistent B-ALL	26.1	0.4

*ALL* acute lymphoblastic leukemia, *AML* acute myeloid leukemia, *Bina* Blinatumomab, *CART* Chimeric antigen receptor T-cell therapy, *Chemo* chemotherapy, *CML* chronic myeloid leukemia, *CP* chronic phase, *CR* complete remission, *DOD* die with disease, *Dx* diagnosis, *Dz* disease, *FC* flow cytometry, *F/U* follow-up, *Ino* Inotuzumab, *LBL* lymphoblastic lymphoma, *LyBP* lymphoid blast phase, *MMR* major molecular remission, *Mono* monoblastic or monocytic morphology, *MRC* myelodysplasia-related changes, *MS* myeloid sarcoma, *MyBP* myeloid blast phase, *NA* not available, *Ofa* Ofatumumab, *OS* overall survival, *Pt* patient, *R* Rituximab, *SCT* allogeneic stem cell transplant, *TKI* tyrosine kinase inhibitor, *Tx* treatment.

^a^Occult myeloid/lymphoid neoplasm with *JAK2* rearrangement.

^b^Occult myelodysplastic syndrome.

^c^History of myelodysplastic syndrome.

^d^History of polycythemia vera.

^e^History of primary myelofibrosis.

^f^History of chronic myeloid leukemia.

Morphologically, AML, either presenting as the first or the second leukemia, predominantly displayed monocytic or myelomonocytic differentiation (21/33, 64%) (Table 1 and Fig. S1). The disease in the remaining patients was classified as AML with minimal differentiation (5/33, 15.2%), AML with maturation (3/33, 9.1%), pure erythroid leukemia (2/33, 6.1%), AML without maturation (1/33, 3.0%), and MyBP-CML (1/33, 3.0%). The immunophenotype of these cases, as detailed in Table S3, generally aligns with that of their respective typical leukemia types.

Following lineage switch, 28 patients underwent chemotherapy, with eight of them also received HSCT (Table 1); three died shortly without receiving treatment; and treatment specifics were unknown for two. At the last follow-up, 25 patients died and eight were alive. Five of the eight surviving patients achieved CR of both leukemias and three had persistent second leukemia. The median survival for the whole cohort was 12.3 months from the diagnosis of the first leukemia and 2.9 months after lineage switch. Notably, among the eight patients who received HSCT after lineage switch, all seven with available information achieved CR although two subsequently relapsed with the second leukemia. In contrast, only one of 19 patient who didn’t receive HSCT after lineage switch was in CR at last follow-up (*p* < 0.001). HSCT was also associated with a longer overall survival (OS) from the second leukemia (93.7 vs 1.9 months; *p* < 0.001). Furthermore, the univariate Cox analysis showed that HSCT, pediatric status, and the presence of 11q23/*KMT2A* fusions were all correlated with OS (*p* < 0.2), while a multivariate analysis identified HSCT as the sole independent prognostic factor (*p* < 0.001).

A pivotal contribution of our study is the enhanced understanding of the genetic and molecular features of leukemic lineage switch (Fig. 1, Tables S4 and S5). The most common chromosomal alterations shared between the first and second leukemias were 11q23/*KMT2A* fusions (18/33, 54.5%). These rearrangements predominantly arose from t(4;11) (*n* = 11), followed by t(9;11) and t(11;19) (*n* = 2 each), as well as additional individual cases involving t(2;11), t(10;11), and inv(11). Other shared alterations included 5q- in a complex karyotype (*n* = 4), −7/7q- (*n* = 2), +8 in a complex karyotype (*n* = 2), and -9p-/*CDKN2A* deletion (*n* = 2), along with t(12;19)/*TCF3::ZNF384*, t(5;14)/*TCLX3::BCL11b*, t(9;22)/*BCR::ABL1*, 17p11.2 aberrations, and +13, each observed in one case. Notably, the second leukemia exhibited a more complex karyotype compared to the first leukemia, with more cases carrying three or more chromosomal aberrations (17/29 vs 10/30, *p* = 0.05), indicating an evolving genetic landscape.

**Fig. 1:**
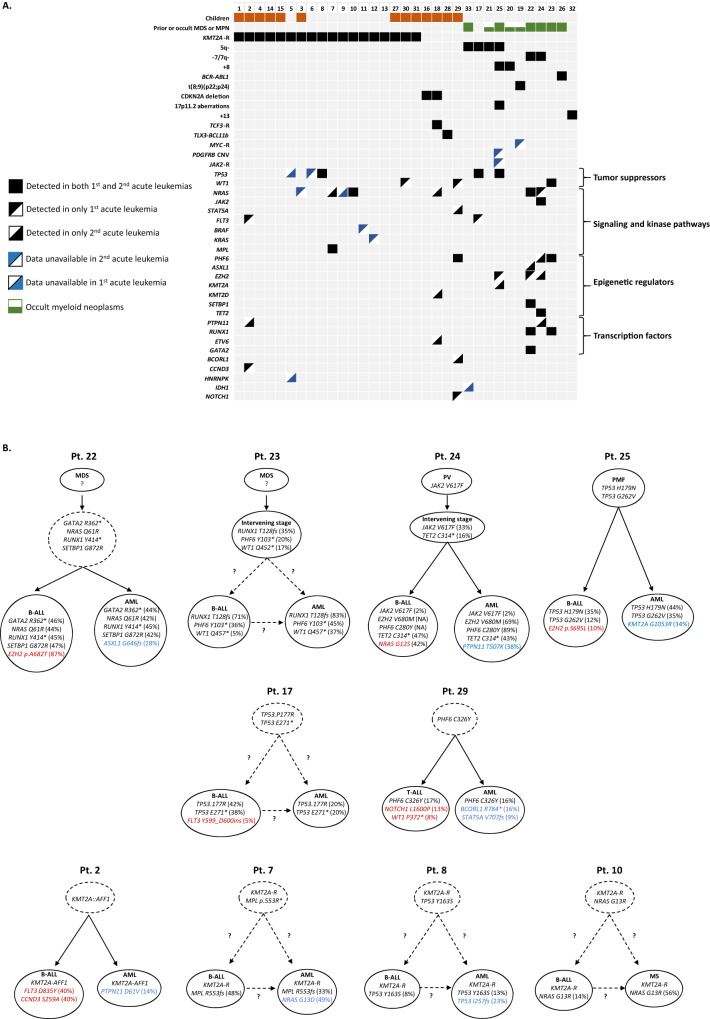
Clonal evolution and mutation analysis in leukemic lineage switch. **A** Key clinical, cytogenetic, and molecular findings in patients with leukemic lineage switch. **B** Clonal architecture of individual patients based on the results of NGS performed on paired specimens of the first and second leukemias. VAF for specific mutations are indicated in parentheses. Dashed circles and dashed lines: inferred clones or pathways without direct molecular evidence. In patient 24, the VAF of *JAK2* V617F in both the first and second leukemias was notably lower compared to other mutations, suggesting a loss of *JAK2* mutation during leukemic transformation of PV, a phenomenon previously reported. Patient 29 was undergoing steroid treatment at the time of NGS for T-ALL, which was conducted one week after the initial diagnosis, with a bone marrow blast count of 49.5% and peripheral blood blasts at 21% by differential count. MDS myelodysplastic syndrome, NGS next-generation sequencing, Pt patient, PV polycythemia vera, VAF variant allele frequency.

Despite the substantial genetic heterogeneity, cases can be simplistically divided into two distinct clinical subgroups based on the presence or absence of 11q23/*KMT2A* fusions (Fig. 1A, Table S1). The most pronounced difference between the two groups lies in the high prevalence of an antecedent chronic myeloid malignancy (CMN) in patients lacking 11q23/*KMT2A* fusions (6/15, 40.0%), with a median interval of 3.5 years (range, 0.2–8 years) preceding the diagnosis of the first acute leukemia. Specifically, the diseases included myelodysplastic syndrome (*n* = 3), polycythemia vera, primary myelofibrosis, and CML (*n* = 1 each). Another three patients likely had either a “subclinical” CMN or clonal hematopoiesis, inferred from the sustained stem-line cytogenetic abnormalities during the interval between the two acute leukemias. Interestingly, despite the presence of antecedent or “subclinical” CMN, B-ALL typically emerged as the initial leukemia (8/9, 88.9%), which, unexpectedly, displayed cytogenetic abnormalities characteristically associated with myeloid neoplasms (7/8, 87.5), including t(8;9)(p22;p24.1) known to involve *PCM1::JAK2*, 5q-, -7/7q-, and +8. Remarkably, no patients with 11q23/*KMT2A* had CMN prior to the first leukemia. Furthermore, compared to patients with *KMT2A* fusions, those without the fusions were older (46.1 vs 25.2 years, *p* = 0.03) with less frequent monocytic differentiation of AML (5/15 vs 16/18, *p* < 0.001). Notably, two patients in the latter group experienced a lineage switch to pure erythroid leukemia, a novel finding not previously reported. Lastly, patients without 11q23/*KMT2A* fusions tended to have a more complex karyotype and more detected mutations, possibly reflecting the founder effect of 11q23/*KMT2A* fusions obviating the need for additional leukemogenic events. Targeted sequencing in 19 patients identified *TP53, NRAS*, and *WT1* as the most frequently mutated genes. Yet, their mutation frequencies in our cohort aligned with those in general leukemias of the same lineage, casting doubt on their driver role in lineage switch [9, 10]. In contrast, mutations of *EZH2* and *RUNX1* occurred at a significantly higher rate (20%) in our B-ALL patients without 11q23/*KMT2A* fusions, in sharp contrast to less than 1% in the general B-ALL population [11, 12]. The finding, despite the small sample size, suggests the importance of monitoring for lineage switch in B-ALL with these mutations. In addition, recurrent alterations in *EZH2* and *KMT2A* hint at the role of epigenic dysfunction in leukemic lineage switch [13]. In support, in vitro research has demonstrated that changes in DNA methylation can trigger a lineage switch in leukemic cells [14]. Other mutated genes broadly fell into four groups: tumor suppressors, signaling and kinase pathways, epigenetic regulators, and transcription factors (Fig. 1A).

In 10 patients, sequencing data were available in both their first and second leukemias (Fig. 1B), which shed important lights on the cellular origin and evolution pathway of lineage switch. In five cases, findings supported divergent clonal evolution: aside from shared genetic alterations, distinct additional mutations were observed between the two leukemias. Hypothetically, in such cases, both leukemias are derived from the same leukemia-initiating cell, which retains the potential to differentiate into either a lymphoid or myeloid lineage. In contrast, the other five patients showed no clear branching in clonal architecture: they maintained the whole set of original mutations, with or without acquiring additional mutations in the second leukemia. Perhaps, in these scenarios, lineage switch originates from the bulk of leukemic blasts through either direct reprogramming or dedifferentiation to a multipotent state followed by commitment to a new lineage. This complexity suggests that multiple tumor evolutionary mechanisms may exist. The distinction between different pathways in individual cases could guide the selection of tailored targeted therapies. Two pivotal questions arise: (1) In tumors following divergent clonal evolution, could simultaneous targeting of both lineages prevent tumor escape? And if so, how can we identify patients for whom the benefits outweigh the side effects of additional treatment? (2) For tumors aligned with the reprogramming or dedifferentiation pathways, might the reduction of extrinsic inducible factors serve as a preventive strategy [15]? It is our hope that this study prompts further inquiries into these critical areas.

In conclusion, the present study provides a comprehensive clinicopathological, genetic, and molecular characterization of this dismal event. Potential risk factors include pediatric patients, 11q23/*KMT2A* fusions, B-ALL with *EZH2* or *RUNX1* mutations, B-ALL emerging after a clonally related CMN phase, or B-ALL carrying genetic abnormalities typically associated with myeloid neoplasms. The presence of these risk factors warrants a thorough immunophenotypic evaluation of multiple cell lineages, particularly following therapy or at relapse, to promptly detect a lineage switch. Currently, judicious consolidation with early allogeneic HSCT could be considered in this subset of patients. Enhanced genomic understanding and insights into clonal evolution can pave the way for innovative preventive and therapeutic strategies against this challenging disease.

### Supplementary information

All Supplemental Figures and Tables

## Data Availability

The data supporting this study’s findings are available from the corresponding author upon reasonable request.

## References

[1] Lamble A, Myers RM, Taraseviciute A, John S, Yates B, Steinberg SM (2022). Preinfusion factors impacting relapse immunophenotype following CD19 CAR T cells. Blood Adv.

[2] Wolfl M, Rasche M, Eyrich M, Schmid R, Reinhardt D, Schlegel PG (2018). Spontaneous reversion of a lineage switch following an initial blinatumomab-induced ALL-to-AML switch in MLL-rearranged infant ALL. Blood Adv.

[3] Lucero OM, Parker K, Funk T, Dunlap J, Press R, Gardner RA (2019). Phenotype switch in acute lymphoblastic leukaemia associated with 3 years of persistent CAR T cell directed-CD19 selective pressure. Br J Haematol.

[4] Haddox CL, Mangaonkar AA, Chen D, Shi M, He R, Oliveira JL (2017). Blinatumomab-induced lineage switch of B-ALL with t(4:11)(q21;q23) KMT2A/AFF1 into an aggressive AML: pre- and post-switch phenotypic, cytogenetic and molecular analysis. Blood Cancer J.

[5] Higuchi Y, Tokunaga K, Watanabe Y, Kawakita T, Harada N, Yamaguchi S (2016). Lineage switch with t(6;11)(q27;q23) from T-cell lymphoblastic lymphoma to acute monoblastic leukemia at relapse. Cancer Genet.

[6] Li T, Chen H, Zhang Y, Zeng Y, Zheng L (2021). Very rare lineage switch from acute myeloid leukemia with BCR-ABL1 to B-lymphoblastic leukemia. Ann Hematol.

[7] Fournier E, Inchiappa L, Delattre C, Pignon JM, Danicourt F, Bemba M (2019). Increased risk of adverse acute myeloid leukemia after anti-CD19-targeted immunotherapies in KMT2A-rearranged acute lymphoblastic leukemia: a case report and review of the literature. Leuk Lymphoma.

[8] Zhou T, Wang HW (2021). Antigen loss after targeted immunotherapy in hematological malignancies. Clin Lab Med.

[9] Papaemmanuil E, Gerstung M, Bullinger L, Gaidzik VI, Paschka P, Roberts ND (2016). Genomic classification and prognosis in acute myeloid leukemia. N Engl J Med.

[10] Ho PA, Zeng R, Alonzo TA, Gerbing RB, Miller KL, Pollard JA (2010). Prevalence and prognostic implications of WT1 mutations in pediatric acute myeloid leukemia (AML): a report from the Children’s Oncology Group. Blood.

[11] Penther D, Preudhomme C, Talmant P, Roumier C, Godon A, Mechinaud F (2002). Amplification of AML1 gene is present in childhood acute lymphoblastic leukemia but not in adult, and is not associated with AML1 gene mutation. Leukemia.

[12] Schafer V, Ernst J, Rinke J, Winkelmann N, Beck JF, Hochhaus A (2016). EZH2 mutations and promoter hypermethylation in childhood acute lymphoblastic leukemia. J Cancer Res Clin Oncol.

[13] Chan AKN, Chen CW (2019). Rewiring the epigenetic networks in mll-rearranged leukemias: epigenetic dysregulation and pharmacological interventions. Front Cell Dev Biol.

[14] Bueno-Costa A, Pineyro D, Soler M, Javierre BM, Raurell-Vila H, Subirana-Granes M (2020). B-cell leukemia transdifferentiation to macrophage involves reconfiguration of DNA methylation for long-range regulation. Leukemia.

[15] Wei J, Wunderlich M, Fox C, Alvarez S, Cigudosa JC, Wilhelm JS (2008). Microenvironment determines lineage fate in a human model of MLL-AF9 leukemia. Cancer Cell.

