# Supplementary material for: Genetics and pathologic landscape of lineage switch of acute leukemia during therapy
Source: Blood Cancer J. 2024 Jan 25;14(1):19. doi: 10.1038/s41408-024-00983-2 (PMC10810851; doi:10.1038/s41408-024-00983-2)

Supplemental Table S1. Comparison of clinicopathologic features of patients with lineage-switched leukemia according to *KMT2A* status.

	***KMT2A* +**			***KMT2A*-**		
	**Adult**	**Children**	**Total**	**Adult**	**Children**	**Total**
Number	9	9	18	11	4	15
Acute #1 phenotype						
B-ALL	9	6	15	8	2	10
T-ALL	0	1	1	0	2	2
CML	0	0	0	1	0	1
AML	0	2	2	2	0	2
History of preceding myeloid malignancies	0	0	0	9	0	9
Interval from acute #1 to #2 (m)	7.4	9.8	8.0	6.0	7.3	7.3
HSCT after LS	2	5	7	1	0	1
Median OS from acute #2 (m)	5.3	Not achieved	2.6	1.1	2.0	1.7

Abbreviations: ALL, acute lymphoblastic leukemia; AML, acute myeloid leukemia; CML, chronic myeloid leukemia; HSCT: allogeneic hematopoietic stem cell transplant; LS, lineage switch; m, month; OS, overall survival.

Supplemental Table S2. Treatments of patients with lineage-switching leukemia

Case #	Dx of acute #1	Chemotherapy for acute #1	SCT for acute #1	Dx of acute #2	Chemotherapy for acute #2	SCT for acute #2
1	B-ALL	POG 9407	No	AML, Mono	Clofaribine; idarubicin/cladaribine and clofaribine	No
2	B-ALL	AALL0631: Prednisone/ Vincristine/ Daunorubicin/Erwinia/ blinatumomab	No	AML, Mono	High dose cytarabine/etoposide	No
3	B-ALL	TINI study (Total Therapy for Infants with ALL I, St. Jude Children's Research Hospital)	No	AML, Mono	clofarabine/cytarabine	No
4	B-ALL	Standard induction and maintenance enrolled on study AALL1821 Group1 and received pre-immunotherapy cytoreduction with dexamethasone and Vincristine; CAR-T	No	AML, M1	Induction and re-induction with AML directed therapy per AAML0531 with ADE + GO	No
5	B-ALL	Pediatric inspired induction, followed by consolidation chemotherapy on CALGB 104030 off study with Daunorubicin and vincristine	Yes	AML, Mono	7+3 induction	No
6	B-ALL	R-hyperCVAD; Decadron, Cytarabine, Rasburicase;<br>mHCVD + Inotozumab + Blinatumomab	Yes	AML, MS, Mono	FIA + Vincristine + Pegasapargase + Venetoclax	No
7	B-ALL	vincristine, daunorubicin, asparaginase, corticosteroids, and cytarabine; Blinatumomab	No	AML, M4	Multiple lines of salvage treatment	No
8	B-ALL	R-hyperCVAD; miniVAD + inotuzumab, blinatumomab;<br>FIA + pegasparaginase + vincristine + blinatumomab	No	AML, Mono	CLIA + venetoclax;<br>azacitidine + Ipilimumab + Nivolumab	No
9	B-ALL	Hyper-CVAD + blinatumomab	No	AML, Mono	decitabine; FIA + venetoclax	Yes
10	B-ALL	HyperCVAD;<br>FIA + pegasrargase + blinatumomab	Yes	MS, Mono	Fludarabine+Cytarabine+ mylotarg	No
11	B-ALL	Enrolled in clinical trial, E1910 with blinatumomab;<br>off trial and treated with Inotuzumab	No	AML, M4	7+3 induction	No
12	B-ALL	Hyper-CVAD, ADCT-402	No	AML, mono	No treatment	No
13	B-ALL	Hyper-CVAD	No	AML, mono	FLAG-Ida plus venetoclax	Yes
14	B-ALL	AALL1131, VHR arm for CNS3; then switched to infant protocol due to presence of MLL--> TAT AALL0631 arm C	No	AML, Mono	Idarubicin/cladribine; azacytidine + fludarabine/cytarabine	Yes
15	B-ALL	AALL15P1 protocol; HD MTX and HD Arac, plus Triple ITT;<br>CD19-targeted CAR-T (Kymriah)	No	AML, Mono	Gemtuzumab with Fludarabine and HD Cytarabine;	Yes
16	B-ALL	AALL0331	No	AML, Mono	AAML0531 (daunomycin, etoposide, cytarabine)	No
17	B-ALL	Mini-HCVD + Inotuzumab + rituximab+ Blinatumomab	No	AML, M0	Azacytidine, venetoclax, APR246	No
18	B-ALL	Inotuzumab, vincristine, daunorubicin, and IT MTX	N0	AML, Mono	Cytarabine, Daunorubicin, Etoposide	No
19	B-ALL	CODOX-M + IVAC with Rituximab	No	AML, M4	7+3, FLAG, Azacitadine + Venetoclax, Nivo	Yes
20	B-ALL	CALGB10102, intrathecal methotrexate;<br>Etoposide chemotherapy and total-body irradiation	Yes	AML, Mono	7+3 induction	No
21	B-ALL	CALGB19802	NA	AML, M6	No treatment	No
22	B-ALL	CALGB 10403 AYA	Yes	AML, M2 (MRC)	HMA, venetoclax	No
23	B-ALL	Mini-HCVD + rituximab + blinatumomab		AML, M2	Decitabine	No

24	B-ALL	Mini-HCVD + Ofatumumab	No	AML, M2	No treatment	No
25	B-ALL	Vincritine, rituximab, cytarabine, MTX, blinatumomab	No	AML, M6	NA	NA
26	B-LyBP	Hyper-CVAD, Gleevec, MTX, cytarabine	No	MyBP	lonafarnib, Gleevec	No
27	T-ALL	AALL0434	No	AML, M0	St Jude AML-O2 protocol	Yes
28	T-ALL	Prednisone, vincristine, daunorubicin, pegylated asparaginase, and intrathecal cytarabine and methotrexate. Consolidation and Delayed Intensification: Cyclophosphamide, cytarabine, doxorubicin, dexamethasone and mercaptopurin	No	AML, M4	Unknown salvage regimens	No
29	T-ALL	Dexamethasone, L-asparaginase, indesine; idarubicine; idarubicin + cytarabine	Yes	AML, M0	NA	NA
30	AML, Mono	AAML1031 (arm b bortezomib--> low risk arm)	No	B-ALL	AALL0931	Yes
31	AML, Mono	AML-2002, including cytarabine, etoposide, L-asparaginase	No	B-ALL	Cytarabine, L-asparaginase, MXT	Yes
32	AML, M2	Clofarabine, cytarabine, low-dose Ara-C	No	B-ALL	Unknown regimen	No
33	AML, M0 (MRC)	Clofarabine, cytarabine	No	B-ALL	Clofarabine, cytarabine	No

Supplemental Table S3. Immunophenotype of lineage-switching leukemia

Case #	Immunophenotype of acute #1	Immunophenotype of acute #2
1	Pos: CD15, CD19, CD22, CD34, HLA-DR<br>Neg: CD10, CD11b, CD13, CD14, CD16+56, CD20, CD33, CD42+61, CD71, CD117, GlyA, Kappa, Lambda, T-cell markers (CD3, CD4, CD5, CD7, CD8)	Pos: CD4, CD7, CD11b, CD15, CD33, CD34, CD71, HLA-DR<br>Neg: CD13, CD14, CD16+56, CD42+61, CD117, CD138, GlyA, B-cell markers (CD10, CD19, CD20, CD22, Kappa, Lambda), T-cell markers (CD3, CD5, CD8)
2	Pos: CD15(p), CD19, CD22 (d/v), CD34, CD38(v), CD45(v), CD79a, HLA-DR, TdT<br>Neg: CD10, CD11b, CD13, CD14, CD16, CD20, CD25, CD33, CD36, CD41/CD61, CD56, CD64, CD71, CD117, Kappa, Lambda, MPO, T-cell markers (CD2, CD3, cyCD3, CD4, CD5, CD7, CD8)	Pos: CD2(s/d), CD4(s/d), CD7(s/d), CD11b(v), CD13(d), CD33, CD34(s/d), CD36 (s/d), CD38(b), CD45(m), CD56(v), CD64(b), CD79(s/d), CD117(s), MPO(s/d)<br>Neg: CD3, CD8, CD14, CD16, TdT, and B-cell markers (CD10, CD19, CD20, CD22)
3	Pos: CD9, CD19, CD20(d/p), CD22, CD34, CD38, CD58, CD79a, HLA-DR, TdT<br>Neg: CD10, CD13, CD33, CD25, CRLF2, MPO	Pos: CD9(p), CD13, CD14, CD15, CD33, CD38, CD56(p), CD64, HLA-DR, lysozyme<br>Neg: CD11c, CD117, CD123, and B-cell markers (CD19, CD20)
4	Pos: CD15(p), CD19, CD33(d/p), CD34, cyCD79a, HLA-DR, TdT, Pax5<br>Neg: CD10, CD13, MPO	Pos: CD7(p), CD15(p), CD33(d/p), CD38, CD58, CD64(d/p), CD117, HLA-DR, MPO(ss)<br>Neg: CD9, CD11b, CD11c, CD13, CD14, CD34, CD56, CD123, TdT, and B-cell markers (CD10, CD19, CD20, cyCD22, cyCD79a)
5	Pos: CD19, CD34(s), CD38, CD45(d), cyCD79a, HLA-DR, TDT<br>Neg: CD10, CD11b, CD11c, CD13, CD14, CD15, CD20, cyCD22, CD33, CD56, CD64, CD117, MPO, CRLF2, Kappa, Lambda, T-cell markers (CD1a, CD2, CD3, cyCD3, CD4, CD5, CD7, CD8)	Pos: CD13(s), CD33(p), CD34(s), CD38, CD45(b), CD64(p), CD117(p), HLA-DR, MPO<br>Neg: CD11b, CD11c, CD14, CD15, CD56, B-cell markers (CD10, CD19, CD20, cyCD22, cyCD79a, Kappa, Lambda), T-cell markers (CD1a, CD2, CD3, cyCD3, CD4, CD5, CD7, CD8)
6	Pos: CD19, CD15(p), CD22, CD34(p), CD38, CD58, HLA-DR, TdT<br>Neg: CD9, CD10, CD13, CD20, CD25, CD33, CD56, CD66c, CD117, and T-cell markers (CD2, CD3, CD4, CD5, CD7, CD8)	Pos: CD4(p), CD13, CD15(inc), CD25(p), CD33, CD36, CD38, CD64, CD68, CD123(inc), HLA-DR(p), lysozyme<br>Neg: CD14, CD34, CD56, CD117, MPO, TdT, B-cell markers (CD10, CD19, CD20, CD22, Pax5), T-cell markers (CD2, CD3, cyCD3, CD5, CD7)
7	Pos: CD9, CD15(p), CD19, CD22, CD34(p), CD38, CD45(d), cyCD79a, HLA-DR, TdT<br>Neg: CD10, CD13, CD16, CD20, CD33, CD36, CD56, CD64, CD66c, CD117, MPO, and T-cell markers (CD2, sCD3, cCD3, CD7)	Pos: CD13, CD15, CD33, CD45, CD36, CD56, CD64(p), MPO<br>Neg: CD16, CD34, CD38, CD117, HLA-DR, TdT, B-cell markers (CD10, CD19, cyCD22, cyCD79a), T-cell markers (CD2, CD3, cyCD3, CD7)
8	Pos: CD15(p), CD19, CD22(p), CD33(p), CD38, CD45(d), cyCD79a(d), CD123, HLA-DR(p), TdT(d)<br>Neg: CD10, CD13, CD14, CD20, CD25, CD34, CD36, CD41, CD56, CD64, CD66c, CD117, CRLF2, cyIgM, MPO, T-cell markers (CD2, CD3, cyCD3, CD4, CD5, CD7)	Pos: CD4(p), CD33(p), CD36(p), CD38(b), CD64, CD123, CD117(s), HLA-DR(b), lysozyme<br>Neg: CD2, CD7, CD9, CD10, CD13, CD14, CD19, CD20, CD22, CD24, CD34, CD56, CD66c, CD79a, Pax-5, MPO, TdT
9	Pos: CD19, CD22, CD34, CD33(p), CD38, TdT(p)<br>Neg: CD10, CD13, CD14, CD15, CD20, CD23, CD56, CD117, CD123, HLA-DR, MPO, T-cell markers (CD3, CD4, CD5, CD7, CD8)	Pos: CD4, CD9(d/p), CD15(s), CD25, CD33, CD38, CD56, CD64, CD117(p), CD123, HLA-DR<br>Neg: CD13, CD14, CD34, CD36, CD66c, cIgM, MPO, TdT, B-cell markers (CD10, CD19, CD20, CD22), T-cell markers (cyCD3, CD5, CD7)
10	Pos: CD19(d), CD22(d/p), CD34, CD38, CD45(d), CD56, cyCD79a(d), HLA-DT, TdT<br>Neg: CD4, CD10, CD11c, CD13, CD14, CD20, CD33, CD64, CD117, MPO, T-cell markers (CD2, CD3, CD5, CD7, CD8)	Pos: CD11c(s), CD15(s), CD33(d), CD43, CD45, CD68, lysozyme<br>Neg: CD34, CD117, MPO, TdT, and B-cell markers (Pax5, CD10, CD19, CD20, CD22, CD79a)
11	Pos: CD19, cyCD22, CD34(p), CD38, CD45(d), cyCD79a, HLA-DR, TdT<br>Neg: CD10, CD11b, CD11c, CD13, CD14, CD15, CD20, CD33, CD56, CD64, CD117, CRLF-2, MPO, Kappa, Lambda, T-cell markers (CD1a, CD2, CD3, cyCD3, CD4, CD5, CD7, CD8)	Major monocytic component: positive for CD2, CD7, CD11b, CD14(p), CD33, CD36, CD38, CD45(b), CD64, HLA-DR; negative for CD4, CD5, CD13, CD19, CD22, CD56, CD117, CD123<br>Minor myeloblast component: positive for CD15(p), CD33, CD34, CD38, HLA-DR(p); negative for CD11b, CD13, CD14, CD36, CD56, CD64, CD117, CD123, B-cell markers (CD19, CD22), T-cell markers (CD2, CD4, CD5, CD7)
12	Pos: CD19, CD22(d), CD33(ss), CD34, CD38, CD45(d), cyCD79a, CD123(d), CD123(d), HLA-DR, TdT<br>Neg: CD10, CD13, CD14, CD15, CD20, CD25, CD36, CD41, CD56, CD64, CD66c, CD117, cyIgM, CRLF2, MPO and T-cell markers (CD2, c/s CD3, CD4, CD5, CD7)	Pos: CD4, CD9, CD11c, CD13+33, CD15, CD36(p), CD38, CD56, CD58, CD64, CD68, CD123, HAD-DR(dec), lysozyme<br>Neg: Pax5, CD10, CD14, CD19, CD20, CD22, CD25, CD34, CD66c, CD117, CRLF2
13	Pos: CD19, cCD22, CD22 (d/p), cCD79a, CD45, CD15, HLA-DR, CD38, PAX5+<br>Neg: CD10, CD11c, CD13, CD16, CD20, CD33, CD34, CD36, CD64, CD56, CD117, cMPO, TdT, surface and cyto kappa and lambda, CD2, CD3, cCD3, CD5, CD7, CD23, CD103, CD200.	Pos: Pax5 (variable), CD33, lysozyme<br>Neg: CD19, CD22, CD79a, CD117

14	Positive: CD10, CD19, CD20(h), CD22, CD38, CD45, CD52, CD58, CD99, HLA-DR<br>Negative: CD11b, CD13, CD14, CD15, CD16+56, CD25, CD33, CD34, CD42+61, CD64, CD71, CD117, GlyA, CRLF2, Kappa, Lambda	Pos: CD4(d/p), CD13, CD15(d/p), CD33, CD34(p), CD38, CD45(d), CD58, CD64(d), CD99, CD117<br>Neg: CD11b, CD14, CD16+56, CD25, CD42+61, CD52, CD71, HLA-DR, GlyA, B-cell markers (CD10, CD19, CD20, CD22, Kappa, Lambda), T-cell markers (CD2, CD3, CD5, CD7, CD8)
15	Pos: CD19, CD22, CD34, CD38, CD58, HLA-DR, surface lambda<br>Neg: CD10, CD13, CD15, CD20, CD33, CD56, MPO, TdT, surface Kappa	Pos: CD7 (ss), CD33, CD36, CD64, HLA-DR, MPO<br>Neg: CD13, B-cell markers (CD10, CD19, CD20, CD22, cyCD79a), T-cell markers CD2, CD3, CD4)
16	Pos: CD19, CD22, HLA-DR<br>Neg: CD10, CD11b, CD13, CD14, CD15, CD16+56, CD20, CD33, CD42+61, Kappa, Lambda, T-cell markers (CD3, CD4, CD5, CD7, CD8)	Pos: CD4, CD11b, CD13, CD15, CD16+56, CD33<br>Neg: CD14, CD34, CD42+61, CD71, CD117, CD138, HLA-DR, GlyA, B-cell markers (CD10, CD19, CD20, CD22, Kappa, Lambda), T-cell markers (CD3, CD5, CD7, CD8)
17	Pos: CD10, CD13, CD19, CD20, CD22, CD25, CD33, CD34, CD38, CD38, CD45(d), CD66c, CD71, cyCD79a, CD123, cyIgM, HLA-DR, TdT<br>Neg: CD14, CD15, CD41, CD56, CD64, CD117, CRLF2, MPO, T-cell markers (CD2, CD3, cyCD3, CD4, CD5, CD7)	Pos: CD13(p), CD33, CD34, CD38, CD45(d), CD56(p), CD71(p), CD123(p) CD117(p), HLA-DR(p)<br>Neg: CD15, CD24, CD64, CD66c, MPO, TdT, B-cell markers (CD10, CD19, CD20, CD22), T-cell markers (CD3, cyCD3, CD4, CD5, CD7)
18	Pos: CD9(p), CD19, CD20 (p), CD33(p), CD34, CD38 (p), CD45(d), CD79a(d), CD123, HLA-DR<br>Neg: CD3, CD4, CD7, CD10, CD11b, CD11c, CD14, CD15, cCD22, CD24, CD56, CD64, CD117, MPO, kappa, lambda, TdT	Pos: CD9(p), CD11b, CD15, CD24, CD33, CD38, CD45(m), CD56(p), CD64, CD123(p), HLA-DR, MPO<br>Neg: CD4, CD11c, CD13, CD14, CD34, CD117, TdT, B-cell markers (CD10, CD19, CD20, CD22, cyCD22, cyCD79), T-cell markers (CD2, CD5)
19	Pos: CD10, CD19, CD20(d/-), CD38, CD45(d), CD79a, Pax5<br>Neg: CD3, CD5, CD34, MPO, TdT, BCL2, BCL6, surface Kappa and Lambda	Major component: positive for CD11b(p), CD13(p), CD33(b), CD38, CD64(d), MPO; negative for CD14, CD16, CD34, CD117, B-cell markers (CD19, CD20, CD22, cyCD22, CD79a), T-cell marker CD2, CD3, cyCD3, CD5, CD7)<br>Minor component: positive for CD10(p), CD11b, CD13, CD16, CD33(d), CD38, CD64(d), MPO; negative for CD14, CD34, CD117, B-cell markers (CD19, CD20, CD22, cyCD22, CD79a), T-cell marker CD2, CD3, cyCD3, CD5, CD7)
20	Pos: CD13(p), CD19, CD22, CD33(p), CD34, CD79a, Pax5, TdT<br>Neg: CD3, CD4, CD5, CD7, CD10, CD14, CD20, CD41, CD61, CD64, CD117, MPO, Kappa, Lambda	Pos: CD4, CD11C, CD13, CD14, CD33, CD45, CD56, CD64, HLA-DR<br>Neg: CD34, CD41, CD61, CD117, MPO, B-cell markers (CD10, CD19, CD20, CD22), T-cell markers (CD3, CD4, CD5, CD7)
21	Pos: CD10, CD13(d), CD19, CD22, CD34, CD79a, CD123, HLA-DR, PAX5, TdT<br>Neg: CD14, CD20, CD33, CD41, CD61, CD64, CD117, MPO, Kappa, Lambda, T-cell markers (CD3, CD4, CD5, CD7)	Pos: CD4, CD33, CD34(p), CD71, CD117(p), CD235a, E-cad, GlyA, HLA-DR<br>Neg: CD13, CD41, CD61, MPO, B-cell markers (CD10, CD19, CD20, CD22), T-cell markers (CD3, CD4, CD5, CD7)
22	Pos: CD10, CD13(d/m), CD15(p), CD19, CD20 (d/-), CD22(d), CD25(d), CD34, CD45(d), CD56(d), HLA-DR, TdT<br>Neg: CD200, MPO, Kappa, Lambda	Pos: CD13, CD15, CD33, CD34, CD38(p), CD45, CD117, CD123(d), CD200, HLA-DR, MPO<br>Neg: CD56, TDT, B-cell markers (CD10, CD19, CD20, CD22)
23	Pos: CD4(d), CD13(ss), CD19, CD22, CD25(p), CD34(p), CD36(p), CD38, cyCD79a, HLA-DR, TdT<br>Neg: CD2, CD5, CD10, CD14, CD15, CD33, CD41, CD56, CD63, CD117, CD123, MPO	Pos: CD4(d), CD13, CD25(b), CD33, CD34, CD36(b), CD38(d), CD56(p), CD117(p), CD123, HLA-DR<br>Neg: CD14, CD15, CD41, CD64, CD66c, MPO, B-cell markers (CD10, CD19, CD20, CD22), T-cell marker (CD2, CD3, cyCD3, CD5)
24	Pos: CD19(ss), CD20, CD22, CD34(ss), cyCD79a, HLA-DR, TdT<br>Neg: CD10, CD13, CD14, CD15, CD25, CD33, CD36, CD41, CD56, CD64, CD66c, CD117, CD123, CRLF2, cyIgM, MPO, T-cell markers (CD2, CD3, cyCD3, CD4, CD5, CD7)	Pos: CD13, CD25(p), CD33(d), CD34, CD38, CD117, CD123, HLA-DR, MPO(s), TdT<br>Neg: CD14, CD15, CD36, CD41, CD56, CD64, CD66c, CRLF2, B-cell markers (CD10, CD19, CD22), T-cell markers (CD2, CD3, cyCD3, CD4, CD5, CD7)
25	Pos: CD10, CD19, CD20, CD22, CD79a, TdT<br>Neg: CD3, CD13, CD34, CD117, MPO	Major erythroid component: positive for CD36, CD71(s), CD117(s), CD235, Glycophorin(s), H-ferritin, PAS; negative for CD19, CD34, CD41, CD42, CD61, HLA-DR, MPO, Pax5<br>Minor myeloblast component: positive for CD7(p), CD13, CD33, CD34, CD38(d), CD117(b), HLA-DR; negative for CD2, CD4, CD5, CD19, CD22, CD36, CD56, CD64
26	Pos: CD9, CD10, CD19, cyCD22, CD34, CD38, cyCD79a, cyIgM, HLA-DR, TdT<br>Neg: CD13, CD20, CD33, CD56, CD64, CD117, sIg, MPO, T-cell markers (CD2, CD3, CD5, CD7)	Pos: CD7(p), CD13, CD38, CD117, HLA-DR(p), MPO<br>Neg: CD33, CD34, TdT, B-cell markers (CD10, CD19, CD20)
27	Pos: CD2(s), cyCD3, CD5, CD7, CD38, CD49d, CD56(p), CD117(p), CD183(p)<br>Neg: CD3, CD10, CD13, CD14, CD19, CD20, CD33, CD34, CD41, CD64, HLA-DR, MPO, TdT	Pos: CD7(ss), CD13(p), CD15(s), CD33, CD34, CD38(d), CD56(p), CD117(d), CD123(p)<br>Neg: CD10, CD14, CD19, CD22, CD36, CD41, CD64, HLA-DR, MPO, TdT, and T-cell markers (CD1a, CD2, CD3, cyCD3, CD4, CD5)

28	Pos: cyCD3, CD4, CD5(d), CD7, CD38, cyCD79a, TdT<br>Neg: CD2, sCD3, CD8, CD19, CD56, CD34, HLA-DR, MPO	Pos: CD4, CD11b(v), CD33, CD38, CD56, MPO(b)<br>Neg: CD3, cyCD3, CD5, CD7, CD34, HLA-DR, TdT
29	Pos: CD1a(p), CD2(p), cCD3(p), CD4(d), CD5(d), CD7, CD8(d), CD10, CD38, CD99, TdT<br>Neg: CD2, CD3, CD9, CD13, CD33, CD34, CD56, CD64, CD117, MPO, B-cell markers (CD19, CD22)	Pos: CD7, CD13(p), CD33, CD34, CD38(b), CD45(d), CD56, CD117(dec), CD123, HLA-DR(dec)<br>Neg: CD14, CD15, CD25, CD64, MPO, TdT, B-cell markers (CD19, CD22), T-cell markers (CD2, CD3, CD4, CD5)
30	Pos: CD11b, CD13, CD15, CD16+56, CD19(wk/ss), CD33, CD34, CD38, CD45(d), CD52(p), CD58, CD64, CD71, CD99, HLA-DR<br>Neg: CD10, CD14, CD22, CD25, CD42+61, CD117, GlyA	Pos: CD15, CD19, CD22(p), CD33(p), CD34, CD38, CD45(d), CD58, cyCD79a, CD99, HLA-DR, TdT<br>Neg: CD11b, CD14, CD42+61, CD64, CD117, GlyA, MPO, B-cell markers (CD10, CD20, Kappa, Lambda), and cyCD3
31	Pos: CD4, CD11b, CD15, CD33, CD16+56, HLA-DR<br>Neg: CD13, CD14, CD42+61, B-cell markers (CD10, CD19, CD20, CD22, Kappa, Lambda), T-cell markers (CD3, CD5, CD7, CD8)	Pos: CD19, CD22, CD38, CD45(d), CD79a, HLA-DR, Pax5, TdT(s)<br>Neg: CD11b, CD13, CD14, CD15, CD16+CD56, CD33, CD34, CD36, CD71, CD117, B-cell marker (CD10, CD20, Kappa, lambda), T-cell markers (CD3 , CD4, CD5, CD7, CD8), MPO
32	Pos: CD13, CD15, CD33(p), CD34, CD38, CD117, HLA-DR, MPO(p), TdT<br>Neg: CD14, CD41, CD56, CD64, B-cell markers (CD10, CD19, CD20), T-cell markers (CD2, CD3, cyCD3, CD5, CD7)	Pos: CD9, CD13(s), CD19, CD20, CD22, CD33(ss), CD34, CD38, CD79a, cyIgM, HLA-DR, TdT<br>Neg: CD10, CD14, CD41, CD56, CD64, CD117, MPO, T-cell markers (CD2, CD3, cyCD3, CD5, CD7)
33	Pos: CD13, CD15(p), CD33(p), CD34, CD38, CD117, HLA-DR<br>Neg: CD14, CD41, CD56, CD64, MPO, TdT, B-cell markers (CD10, CD19, CD20, CD22, Kappa, lambda), T-cell markers (CD3, CD5, CD7, CD8)	Pos: CD13(p), CD22(p), CD25, CD34, CD38(p), CD45(d/-), CD49d, CD79a, CD123, CD184, HLA-DR, Pax5, Oct2(s), TdT<br>Neg: CD5, CD7, CD10, CD11c, CD14, CD15, CD19, CD20, CD33, CD56, CD64, CD66c, CD117, cyIgM, lysozyme, MPO

Abbreviations: b, bright; cy, cytoplasmic; d, dim; dec, decreased; d/-, dim to negative; d/p, dim/partial; h, heterogenous; inc, increased; ls, large subset; m, moderate; p, partial; s, subset; ss, small subset; v, variable; wk, weak.

Supplemental Table S4. Cytogenetic and molecular alterations at diagnoses of the first and second acute leukemias

Case #	Karyotypes at Dx of acute #1 and #2	Positive FISH at Dx of acute #1 and #2	Positive mutations at Dx of acute #1 and #2
1	B-ALL: 46,XY,t(4;11)(q21;q23)[19]/46,XY[1]<br>AML: NA	B-ALL: *KMT2A* rearrangement<br>AML: *KMT2A* rearrangement	NA
2	B-ALL: 46,XY,t(4;11)(q21;q23)[15]/46,XY[5]<br>AML: NA	B-ALL: *KMT2A* rearrangement<br>AML: *KMT2A* rearrangement	B-ALL: FLT3 p.D835Y, CCND3 p.S259A, KMT2A::AFF1 [#1]<br>AML: PTPN11 p.D61V, KMT2A::AFF1 [#1]
3	B-ALL: 46,XY,t(4;11)(q21;q23)[20]<br>AML: 46,XY,t(4;11)(q21;q23)[20]	B-ALL: *KMT2A* rearrangement<br>AML: *KMT2A* rearrangement	B-ALL: NRAS p.G12D, NRAS p.Q61K [#2]<br>AML: NA
4	B-ALL: 46,XX,t(4;11)(q21;q23)[2]/46,XX[2]<br>AML: 54,XX,t(4;11)(q21;q23),+der(4)t(4;11)(q21;q23)x3,+6,+7,+13,+21,+22[20]	B-ALL: *KMT2A* rearrangement [%]<br>AML: *KMT2A* rearrangement	NA
5	B-ALL: 46,XX,t(4;11)(q21;q23)[5]/46,XX[8]<br>AML: 61~63<3n>,XXX,-4,der(4)t(4;11)(q21;q23),t(4;11),-5,+6,+7,-9,-11,-12,-13,-14,-15,-16,-17,<br>i(17)(q10),-18,+21,+22,+2~3mar[cp5]/60~64,sl,-X,add(3)(q12),-20,+21,+add(22)(p11.2)[cp8]/<br>61~64,sdl1,-add(3),+add(3)(p21),-5,add(7)(q32),-8,add(9)(p13),-18,+20,-21[cp7]	B-ALL: *KMT2A* rearrangement<br>AML: NA	B-ALL: NA<br>AML: TP53 p. R213*, HNRNPK p. L398del [#3, ?]
6	B-ALL: 46,XY,t(4;11)(q21;q23)[11]/46,idem,add(18)(p11.2)[8]/46,XY[1]<br>AML: 49~51,XY,t(4;11)(q21;q23),-5,del(5)(q22q35),+6,+7,+8,-9,add(12)(p13),+13,-<br>17,add(17)(p11.2),+19,+20,+21,+22,+mar[cp19]	B-ALL: *KMT2A* rearrangement<br>AML: *KMT2A* rearrangement	B-ALL: TP53 p.R282P [#4]<br>AML: NA
7	B-ALL: 46,XX,t(4;11)(q21;q23)[20]<br>AML: 52,XX,t(4;11)(q21;q23),t(5;19)(p11;q11),+6,+7,+8,+8,+13,+19[cp10]/79-80,XXXX,-1,-<br>2,t(4;11)(q21;q23),der(4)t(4;11),-5,-7,-9,-10,-10,-11,-15,-16 -17,-21,-22[cp9]/45,XX,-21[1]	B-ALL: *KMT2A* rearrangement<br>AML: *KMT2A* rearrangement	B-ALL: MPL p.K553Rfs [#5]<br>AML: MPL p.K553Rfs, NRAS p.G13D [#5]
8	B-ALL: 46,XX,t(4;11)(q21;q23)[12]/47,idem+8[2]/46,XX[6]<br>AML: 61~65,XX,-X,+add(1)(p13),-3,der(4)t(4;11)(q21;q23),t(4;11),+del(6)(q15q21),-10,-11,-17, -17,<br>-17,+1~6mar[cp12]/46,XX[8]	B-ALL: *KMT2A* rearrangement<br>AML: *KMT2A* rearrangement	B-ALL: TP53 p.Y163S [#4]<br>AML: TP53 p.Y163S, TP53 p.I257fs [#6]
9	B-ALL: 46,XY,t(4;11)(q21;q23)[20]<br>AML: 84~87,XXYY,-1,der(4)t(4;11)(q21;q23),t(4;11),-5,-10,-11,-12[cp20]	B-ALL: *KMT2A* rearrangement<br>AML: 3' deletion of *KMT2A*	B-ALL: KMT2A::AFF1<br>AML: NRAS p.Q61H [#4]
10	B-ALL: 46,XX,t(4;11)(q21;q23)[20]<br>MS: NA	B-ALL: *KMT2A* rearrangement<br>MS: *KMT2A* rearrangement	B-ALL: NRAS p.G13R [#7]<br>MS: NRAS p.G13R [#8]
11	B-ALL: 46,XX,t(4;11)(q21;q23) [5]/46,XX[15]<br>AML: 46,XX,t(4;11)(q21;q23)[15]/46,XX[5]	B-ALL: *KMT2A* rearrangement<br>AML: NA	B-ALL: BRAF p.L692S [#3, ?]<br>AML: NA
12	B-ALL: 46,XX,t(11;19)(q23;p13.3)[14]/46,XX[6]<br>AML: 46,XX,+i(8)(q10),t(11;19)(q23;p13.3),-16[12]/46,XX,+i(8)(q10),t(11;19),add(14)(p11.2),-<br>16[cp6]/47,XX,+X,t(11;19)[2]	B-ALL: *KMT2A* rearrangement<br>AML: *KMT2A* rearrangement	B-ALL: KRAS p.G12D [#7]<br>AML: NA
13	B-ALL: 46,XX,t(11;19)(q23;p13.3)[13]/46,XX[7]<br>AML: 46,XX,t(11;19)(q23;p13.3)[16]/46,XX[4]	B-ALL: *KMT2A* rearrangement<br>AM: NA	B-ALL: NA<br>AML: None [#5]
14	B-ALL: 46,XY,add(7)(p22),t(7;8)(q33-35;q24),der(9)inv(11)(q21q23)t(9;11)(p22;q23)<br>del(9)(q13q34),der(11)inv(11)t(9;11)[18]/46XY[2]<br>AML: 46,XY,t(9;11)(p22;q23)[12]/46,XY[8]	B-ALL: KMT2A rearrangement<br>AML: NA	NA
15	B-ALL: NA<br>AML: 46,XY,t(10;11)(p13;q23)[3]/46,XY[17]	B-ALL: *KMT2A* rearrangement<br>AML: *KMT2A* rearrangement	NA
16	B-ALL: 45,X,-Y,del(9)(p13)[3]/46,XY[5]<br>AML: 45,X,-Y,del(9)(p13)[17]/90,idemx2[2]/46,XY[1]	B-ALL: 9p21 (*CDKN2A*) deletion<br>AML: 9p21 (*CDKN2A*) deletion	NA
17	B-ALL: 47,XX,del(5)(q13q33),+add(22)(q13)[12]/45~48,idem,add(7)(q32,del(20)(q11.2q13.3),-21[cp7]<br>AML: 45~46,XX,del(1)(p32),del(5)(q13q33),der(11)hsr(11)(q23)add(11)(q24),-15,add(16)(q13),-17,-21,<br>+add(22)(q13),del(22)(q12),+1~2mar[cp3]	B-ALL: 5q-<br>AML: 5q-	B-ALL: TP53 p.P177R, TP53 p.E271*, FLT3<br>p.Y599_D600insVLPDFREYEY [#4]<br>AML: TP53 p.P177R, TP53 p.E271* [#4]
18	B-ALL: NA<br>AML: 45,XY,add(1)(p36.1),-10,t(12;19)(p13;p13.3)[14]/47,idem,+10,+20[3]/46,XY[3]	B-ALL: *TCF3* rearrangement, homozygous loss of *CDKN2A*<br>AML: *TCF3* rearrangement, homozygous loss of *CDKN2A*, gain of D7S486/7q	B-ALL: TCF3::ZNF384<br>AML: TCF3::ZNF384, NRAS pG12D, KMT2D p.C823*, KMT2D p.E5240*, ETV6 p.F357_I358insLF [#9]

19	B-ALL: 46,XY,+1,dic(1;3)(p12;p25),t(8;9)(p22;p24.1),der(9)t(8;9)(p22;p24.1),t(8;22)(q24.2;q11.2)[17]/ 46,XY[3]<br>Subclinical myeloid/lymphoid neoplasm: 46,XY,t(8;9)(p22;p24.1)[11]/46,XY[9]<br>AML: 46,XY,t(8;9)(p22;p24)[19]/ 46,XY[1]	B-ALL: *IGL::MYC* rearrangement<br>AML: NA	B-ALL: BRCA2 p.E1482*, CDKN2A p16INK4a R80* and p14ARF P94L<br>AML: None
20	B-ALL: 45,X,–Y,der(10)t(5;10)(q26;q13)[4]/46,idem,+13 [7]/46,XY[9]<br>"subclinical" MDS: 47,XY,+8[1]/46,XY[19]<br>AML: 46,X,–Y,t(1;18)(q12;q21.1),t(4;21)(q12;q22),add(7)(p22),+8,der(10)t(5;10) (q26;q13), del(11)(q14q23),del(14)(q24q32),add(17)(q25)[20]	B-ALL: trisomy 8<br>"subclinical" MDS: trisomy 8<br>AML: trisomy 8; additional copy of 5q31 (EGR1)	NA
21	B-ALL: 46,XY,del(5)(q22q35)[16]/39,idem,–3,–7,–9,–15,–16, –19,–20[3]/71,idem×2[cp2]<br>Subclinical MDS: 46,XY,del(5)(q22q35)[16]/46,XY[4]<br>AML: NA	B-ALL: loss of 5q<br>Sublinical MDS: loss of 5q<br>AML: loss of 5q	NA
22	MDS: 45,XY,-7<br>B-ALL: 45,XY,-7[5]/46,XY[15]<br>AML: 45,XY,-7[10]/45,idem,del(5)(q15q33)[3]/45,idem,del(5)(q15q33),add(10)(q22)[3]/46,XY[4]	B-ALL: NA<br>AML: monosomy 7	MDS: NA<br>B-ALL: GATA2 p.R362*, NRAS p.Q61R, RUNX1 p.Y414*, SETBP1 p.G872R, EZH2 p.A682T [#10]<br>AML: GATA2 p.R362*, NRAS p.Q61R, RUNX1 p.Y414*, SETBP1 p.G872R, ASXL1 p.G646fs [#10]
23	B-ALL: 46,XX[20]<br>MDS: 46,XX[20]<br>AML: 47,XX,+8[2]/46,XX[18]	NA	B-ALL: RUNX1 p.T128fs, PHF6 p.Y103*, WT1 p.Q457* [#4]<br>MDS: RUNX1 p.T128fs, PHF6 p.Y103*, WT1 p.Q457* [#4]<br>AML: RUNX1 p.T128fs, PHF6 p.Y103*, WT1 p.Q457* [#4]
24	PV: NA<br>B-ALL: 46,XY,del(7)(q22q34)[cp2]/45,XY,-7[1]/46,XY,-9,+mar[1]/46,XY[16]<br>MDS: 46,XY[20]<br>AML: 45,XY,-7,del(13)(q12q22)[15]/45~50,idem,+8,+13,+19,+21,+mar[cp5]	PV: NA<br>B-ALL: monosomy 7 or 7q-<br>MDS: NA<br>AML: NA	PV: JAK2 p.V617F<br>B-ALL: JAK2 p.V617F, TET2 p.C314*, EZH2 p.V680M, PHF6 p.C280Y, NRAS p.G12S [#4]<br>MDS: JAK2 p.V617F, TET2 p.C314*<br>AML: JAK2 p.V617F, TET2 p.C314*, EZH2 p.V680M, PHF6 p.C280Y, PTPN11 p.T507K [#4]
25	PMF: 46,XY,del(5)(q31q35),add(7)(q11.2),+8,del(11)(q13),del(12)(p11.2),-14,add(15)(q26)[14] /46,XY[6]<br>B-ALL: 46,XY,del(5)(q31q35),add(7)(q11.2),+8,del(11)(q13),del(12)(p11.2),-14,add(14)(q32), add(15)(q26),add(17)(p11.2)[11]/46,idem,add(7)(q22)[3]/46,XY[6]<br>AML: 45~50,XY,del(5)(q31q35),add(7)(q11.2),+8,del(11)(q13),del(12)(p11.2),-14,add(14)(q32), add(15)(q26),add(17)(p11.2),+2~4mar[13]	PMF: NA<br>B-ALL: NA<br>AML: gain of *PDGFRB* and *JAK2*	PMF: TP53 p.H179N, TP53 p.G262V [#7]<br>B-ALL: TP53 p.H179N, TP53 p.G262V, EZH2 p.S695L [#7]<br>AML: TP53 p.H179N, p.G262V, KMT2A p.G1053R [#4]
26	B-LyBP: 46,XY,t(9;22)(q34;q11.2)[9]/46,idem,del(13)(q12q13)[11]/48,idem,+der(22)t(9;22),+mar[2]<br>MyBP: 48,XY,-7,t(9;22)(q34;q11),+21,+21,+mar[20]	B-LyBP: *BCR-ABL1* rearrangement<br>MyBP: NA	B-LyBP: b3a2<br>MyBP: b3a2
27	T-ALL: NA<br>AML: 46,XY,inv(11)(q21q23)x2[19]/46,XY[1]	T-ALL: *KMT2A* rearrangement<br>AML: *KMT2A* rearrangement	NA
28	T-ALL: 46,XY,t(5;14)(q35;q32),del(9)(p21.3),del(13)(q14.11q21.1)[7]/ 46,idem,del(7)(p1?3p?21)[6]/45,idem,dic(7;8)(p1?3;p11.2),del(10)(q22)[6]<br>AML: 67,XY,+Y,der(4)del(4)(q13.1)inv dup(4)(p12pter),del(7)(p11.2p14.1),+8,-9,del(9)(p21.3),-10,-11,-14,-15,der(15) t(11;15)(q13.1;q26),+19,+22[9]	T-ALL: NA<br>AML: *TLX3::BCL11b*	NA
29	T-ALL: 46,XX[20]<br>AML: 46,XX[6]//46,XY[14]	T-ALL: NA<br>AML: NA	T-ALL: PHF6 p.C326Y, NOTCH1 p.L1600P, WT1 P372* [#11]<br>AML: PHF6 p.C326Y, BCORL1 p.R784*, STAT5A p.V707fs [#4]
30	AML: 46,XX,t(2;11)(q11.2;q23)[5]/46,idem,add(14)(q32),t(16;17)(p13.3;q11.2)[5]/46,XX[15]<br>B-ALL: 46,XX,t(2;11)(q11.2;q23)[5]/46,idem,add(14)(q32),t(16;17)(p13.3;q11.2)[5]/46,XX[10]	AML: *KMT2A* rearrangement<br>B-ALL: *KMT2A* rearrangement	NA
31	AML: 46,XY,add(12)(p11.2)[9].ish ins(9;11)(p22;q23q23)(5'MLL+,9cen+,WCP11-; MLL+, WCP11+) /46,XX[15] nuc ish 11q23(MLLx2)(5'MLL spx1)[124]/11q23(MLLx2)[76]<br>B-ALL: 46,XY,t(14;19)(q32;p13).ish t(14;19)(q32;p13)(3'IGH+,5'IGH con 3'IGH+)[7]/46,XY[23] nuc ish (5'MLL x3,3'MLL x2)(5'MLL con 3'MLL x2)[45]/(MLL x2)[155]/12p13(TELx2), 21q22(AML1x2)[200]	AML: *KMT2A* rearrangement<br>B-ALL: *KMT2A* rearrangement; *IGH* gene rearrangement	NA
32	AML: 47,XY,+13[13]/46,XY[7]<br>B-ALL: 47,XY,+13[20]	NA	NA
33	AML: 91-93,XXYY,add(5)(q23),del(9)(?q32q34),-10,+12,+22[cp7]/46,XY[13]<br>B-ALL: 91~93,XXYY,add(5)(q23),del(6)(q21),-7,del(9)(q13q22),-11,+12,+15,-16,del(17)(p11.2), i(17)(q10),-18,+20,+21, +22,+1~4 mar[cp9]/46,XY[11]	AML: loss of 5q31 (*EGFR1*)<br>B-ALL: loss of 5q31 (*EGFR1*)	AML: NA<br>B-ALL: IDH1 R132H [#6]

Abbreviations: ALL, acute lymphoblastic leukemia; AML, acute myeloid leukemia; Chemo, chemotherapy; FISH, fluorescence in situ hybridization; LBL, lymphoblastic lymphoma; LyBP, lymphoid blast phase; MS, myeloid sarcoma; MyBP, myeloid blast phase; NA, not available, not performed, or performed but negative.

[%] Chromosomal banding analysis performed on treated specimen.
[?] Likely pathogenic.
[#1] Tested in UTSW 1,505-gene panel and RNA fusion panel.
[#2] Tested in OHSU 220-gene panel.
[#3] Tested in Kansas 141-gene panel
[#4] Tested in MDACC 81-gene panel.
[#5] Tested in Mayo OncoHeme 42-gene panel.
[#6] Tested by selective targeting sequencing.
[#7] Tested in MDACC 28-gene panel.
[#8] Tested in MDACC solid tumor 50-gene panel.
[#9] Tested in UTSW 1,505-gene panel and RNA fusion panel.
[#10] Tested in Emory 75-gene panel.
[#11] Tested in Daopei 58-gene panel.
[#32] The evidence for clonal relatedness was primarily based on the presence of trisomy 13, which is a relatively nonspecific finding and thus introduces some uncertainty.

Supplemental Table S5: NGS panels

University of Texas Southwestern Medical Center 1,505-gene Panel:
https://www.utsouthwestern.edu/sites/genomics-molecular-pathology/assets/comprehensive_pan-cancer_next_generation_sequencing_solid_tumor_panel_aberration_list.pdf

Oregon Health and science University 220-gene Panel:
ABL1, AKT1, ANKRD26, ARID1A, ARID1B, ASXL1, ASXL2, ATG2B, ATM, ATRX, BCL2, BCL6, BCOR, BCORL1, BIRC3, BIRC6, BLM, BRAF, BRCA1, BRCA2, BRCC3, BRD4, BTK, CALR, CARD11, CASP10, CBL, CBLB, CBLC, CCND1, CCND3, CCR4, CD27, CD79A, CD79B, CDH11, CDKN2A, CDKN2C, CEBPA, CHD2, CHEK2, CREBBP, CRLF2, CSF1R, CSF3R, CTCF, CTLA4, CUX1, CXCR4, DAXX, DDX41, DDX54, DHX15, DHX29, DIS3, DNAH5, DNAH9, DNAJC21, DNM2, DNMT1, DNMT3A, DOCK8, DTX1, EED, EFTUD1, EGFR, ELANE, EP300, ERBB4, ETNK1, ETV6, EZH2, FAM47A, FAM5C, FAS, FAT1, FAT4, FBXO11, FBXW7, FLT3, FOXO1, FYN, GATA1, GATA2, GATA3, GNA13, GNAS, GNB1, GSKIP, HAX1, HIST1H1E, HNRNPK, HRAS, HVCN1, ID3, IDH1, IDH2, IGLL5, IKZF1, IKZF3, IL7R, IRF4, JAK1, JAK2, JAK3, KDM6A, KIT, KLF2, KLHL6, KMT2A, KMT2C, KMT2D, KRAS, LLGL2, LRRC4, LUC7L2, MAGT1, MAML1, MAP2K1, MECOM, MED12, MEF2B, MGA, MLH1, MPL, MSH2, MSH6, MYC, MYD88, NAF1, NBN, NF1, NFKBIE, NOTCH1, NOTCH2, NPAT, NPM1, NRAS, NT5C2, NXF1, PAX5, PCLO, PDGFRA, PHF6, PIGA, PIK3CD, PIM1, PLCG1, PLCG2, PMS2, POT1, PPM1D, PRDM1, PRKCB, PRPF40B, PRPF8, PRPS1, PSMB5, PTCH1, PTEN, PTPN11, RAD21, RB1, RBBP6, RELN, RHOA, RIT1, RPS15, RTEL1, RUNX1, RYR1, RYR2, SAMD9, SAMD9L, SAMHD1, SBDS, SETBP1, SETD2, SETDB1, SF1, SF3A1, SF3B1, SH2B3, SMARCA2, SMARCB1, SMC1A, SMC3, SOCS1, SPEN, SPI1 SRP72, SRSF2, STAG2, STAT3, STAT5B, STXBP2, SUZ12, SYK, SYNE1, TBL1XR1, TCF3, TCF4, TERC, TERT, TET2, TNFAIP3, TNFRSF14, TP53, TRAF3, U2AF1, U2AF2, UBR5, USH2A, VAV1, WAS, WHSC1, WT1, XPO1, ZBTB7A, ZRSR2

University of Virginia Customized FusionPlex Pan-Heme 149-gene Panel:
ABL1, ABL2, AICDA, ALK, ASB13, BATF3, BCL11B, BCL2, BCL2A1, BCL3, BCL6, BCR, BIRC3, BLNK, BMF, BMP7, CBFB, CCDC50, CCND1, CCND2, CCND3, CD274, CD44, CDC25A, CDK6, CDKN2A, CDKN2B, CEBPA, CEBPD, CEBPE, CEBPG, CHD1, CHIC2, CHMP2A (control), CIITA, CREB3L2, CREBBP, CRLF2, CSF1R, CTLA4, CYB5R2, DEK, DENND3, DLEU1, DNMT3B, DNTT, DUSP22, E2F2, EBF1, EIF4A1, ENTPD1, EPOR, ERG, ETV6, EXOC2, FAM216A, FGFR1, FLT3, FOXP1, FUT8, GLIS2, GPI (control), HOXA10, HOXA9, ID4, IKZF2, IKZF3, IL16, IRF4, IRF8, ITPKB, JAK2, KAT6A, KIAA0101, KLF2, KMT2A, LIMD1, LMO1, LMO2, LRMP, LYL1, LZTS1, MAL, MALT1, MAML3, MECOM, MKL1, MLF1, MLLT10, MLLT4, MME, MUC1, MYBL1, MYC, MYH11, NEK6, NF1, NFKB1, NFKB2, NME1, NOTCH1, NTRK3, NUP214, NUP98, P2RY8, PAG1, PAICS, PAX5, PBX1, PDCD1, PDCD1LG2, PDGFRA, PDGFRB, PICALM, PIM1, PIM2, PML, PPAT, PRDM16, PRKAR2B, PTK2B, PTPN1, PYCR1, RAB29, RARAB7A (control), RAG1, RAG2, RANBP1, RARA, RBM15, ROS1, RUNX1, RUNXT1, SIPR2, SEMA6A, SERPINA9, SETD2, SH3BP5, SOX11, STIL, STRBP, TAL1, TCF3, TFG, TLX1, TLX3, TNFRSF13B, TNFSF4, TP63, TYK2, VCP (control), WT1, ZCCHC6

University of Kansas 141-gene Panel:
ABL1, ADA, ANKRD26, ASXL1, ASXL2, ATM, ATRX, BCL6, BCOR, BCORL1, BCR, BIRC3, BLM, BRAF, BRCA1, BRCA2, BRINP3, C17orf97, CALR, CARD11, CBL, CBLB, CBLC, CDKN2A, CEBPA, CHEK2, CREBBP, CRLF2, CSF1R, CSF3R, CTCF, CUX1, DAXX, DDX41, DNM2, DNMT1, DNMT3A, EED, EGFR, ELANE, EP300, ETNK1, ETV6, EZH2, FAM154B, FAM47A, FAS, FBXW7, FLRT2, FLT3, GATA1, GATA2, GJB3, GNAS, HNRNPK, HRAS, IDH1, IDH2, IKZF1, IKZF3, IL7R, JAK1, JAK2, JAK3, KAT6A, KCNA4, KCNK13, KDM6A, KDR, KIT, KLHDC8B, KLHL6, KMT2A, KMT2C, KRAS, LRRC4, LUC7L2, MAP2K1, MLH1, MPL, MSH2, MSH6, MYC, MYD88, NBN, NF1, NOTCH1, NPAT, NPM1, NRAS, NSD1, NTRK3, OR13H1, OR8B12, P2RY2, PAX5, PCDHB1, PDGFRA, PHF6, PML, PMS2, PRAMEF2, PRF1, PRPF40B, PRPF8, PTEN, PTPN11, RAD21, RB1, RELN, RUNX1, SETBP1,

SF1, SF3A1, SF3B1, SH2B3, SH2D1A, SMARCB1, SMC1A, SMC3, SRP72, SRSF2, STAG2, STAT3, STXBP2, SUZ12, TAL1, TERC, TERT, TET2, TNFRSF13B, TP53, TPMT, TUBA3C, U2AF1, U2AF2, WAS, WRN, WT1, XPO1, ZRSR2

Emory University 75-gene Panel:

ABL1, ANKRD26, ASXL1, ATRX, BCOR, BCORL1, BRAF, BTK, CALR, CBL, CBLB, CBLC, CCND2, CDC25C, CDKN2A, CEBPA, CSF3R, CUX1, CXCR4, DCK, DDX41, DHX15, DNMT3A, ETNK1, ETV6, EZH2, FBXW7, FLT3, GATA1, GATA2, GNAS, HRAS, IDH1, IDH2, IKZF1, JAK2, JAK3, KDM6A, KIT, KMT2A, KRAS, LUC7L2, MAP2K1, MPL, MYC, MYD88, NF1, NOTCH1, NPM1, NRAS, PDGFRA, PHF6, PPM1D, PTEN, PTPN11, RAD21, RBBP6, RPS14, RUNX1, SETBP1, SF3B1, SH2B3, SLC29A1, SMC1A, SMC3, SRSF2, STAG2, STAT3, TET2, TP53, U2AF1, U2AF2, WT1, XPO1, ZRSR2

Daopei 58-gene Panel, China:

ALK, ASXL1, ASXL2, BCL2, BRAF, CALR, CARD11, CBL, CCND1, CD79B, CEBPA, CREBBP, CRLF2, CSF3R, CXCR4, DNMT3A, ETV6, EZH2, FBXW7, FLT3, GATA2, ID3, IDH1, IDH2, IL7R, JAK1, JAK2, JAK3, KIT, KRAS, MAP2K1, MEF2B, MPL, MYD88, NOTCH1, NOTCH2, NPM1, NRAS, NT5C2, PAX5, PHF6, PIK3CA, PTEN, PTPN11, RHOA, RUNX1, STBP1, SF3B1, SH2B3, SRSF2, STAT3, TCF3, TET2, TNFAIP3, TP53, U2AF1, WHSC1, WT1

Mayo Clinic OncoHeme 42-gene Panel:

ANKRD26, *ASXL1, BCOR, CALR, CBL, CEBPA, CSF3R, DDX41, DNMT3A, ELANE, ETNK1, ETV6, EZH2, FLT3, GATA1, GATA2, IDH1, IDH2, JAK2, KDM6A, KIT, KRAS, MPL, NPM1, NRAS, PHF6, PTPN11, RAD21, RUNX1, SETBP1, SH2B3, SF3B1, SRP72, SMC3, SRSF2, STAG2, TERT, TET2, TP53, U2AF1, WT1,* and *ZRSR2*

MD Anderson 81-gene Panel:

ANKRD26, ASXL1, ASXL2, BCOR, BCORL1, BRAF, BRINP3, CALR, CBL, CBLB, CBLC, CEBPA, CREBBP, CRLF2, CSFR3, CUX1, DDX41, DNMT3A, EED, ELANE, ETNK1, ETV6, EZH2, FBXW7, FLT3, GATA1, GATA2, GFI1, GNAS, HNRNPK, HRAS, IDH1, IDH2, IKZF1, IL2RG, IL7R, JAK1, JAK2, JAK3, KDM6A, KIT, KMT2A, KRAS, MAP2K1, MPL, NF1, NOTCH1, NPM1, NRAS, PAX5, PHF6, PIGA, PML, PRPF40B, PTEN, PTPN11, RAD21, RARA, RUNX1, SETBP1, SF1, SF3A1, SF3B1, SH2B3, SMC1A, SMC3, SRSF2, STAG1, STAG2, STAT3, STAT5A, STAT5B, SUZ12, TERC, TERT, TET2, TP53, U2AF1, U2AF2, WT1, and *ZRSR2*

MD Anderson 28-gene Panel:

ABL1, ASXL1, BRAF, DNMT3A, EGFR, EZH2, FLT3, GATA1, GATA2, HRAS, IDH1, IDH2, IKZF2, JAK2, KIT, KRAS, MDM2, MLL, MPL, MYD88, NOTCH1, NPM1, NRAS, PTPN11, RUNX1, TET2, TP53, and *WT1*

MD Anderson Solid Tumor 50-gene Panel:

ABL1, AKT1, ALK, APC, ATM, BRAF, CDH1, CDKN2A, CSF1R, CTNNB1, EGFR, ERBB2, ERBB4, EZH2, FBXW7, FGFR1, FGFR2, FGFR3, FLT3, GNA11, GNAQ, GNAS, HNF1A, HRAS, IDH1, IDH2, JAK2, JAK3, KDR, KIT, KRAS, MET, MLH1, MPL, NOTCH1, NPM1, NRAS, PDGFRA, PIK3CA, PTEN, PTPN11, RB1, RET, SMAD4, SMARCB1, SMO, SRC, STK11, TP53, VHL

Supplemental Figure Legend

Supplemental Figure S1. Representative morphologic and immunophenotypic features of lineage-switched leukemia. Patient #8 was initially diagnosed with B-ALL with t(4;11) (top panel). The blasts were positive for CD19, CD22 (partial), CD79a and CD15 (partial), and negative for CD34 and CD10. The disease later switched to AML with monocytic differentiation (bottom panel) expressing lysozyme, CD64, and CD36 but negative for CD14 and B-cell markers. Abbreviations: B-ALL, B-lymphoblastic leukemia; AML, acute myeloid leukemia.

Supplemental Figure S2. Three hypothesized models for the evolutional pathways of leukemic lineage switch.

Supplemental Figure S1

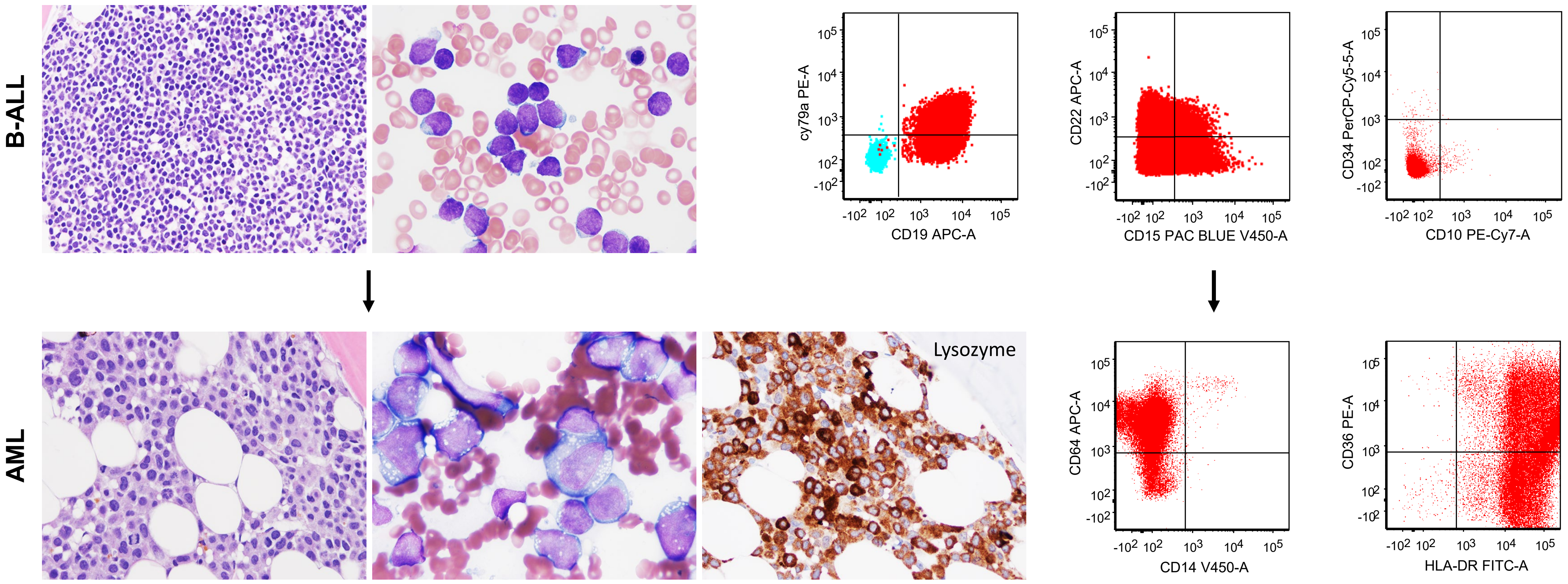

Supplemental Figure S2

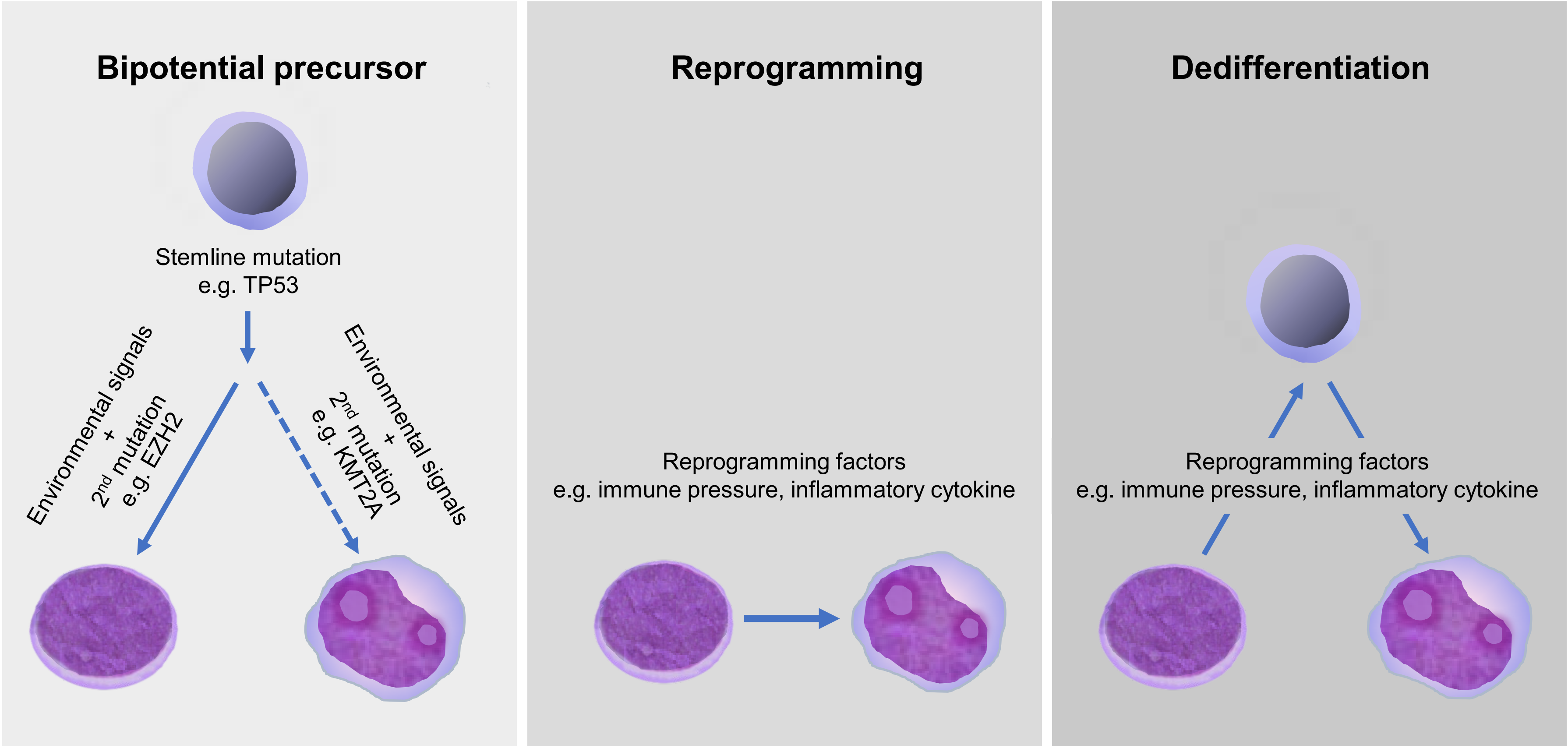

Supplement: Supplementary file 1 — All Supplemental Figures and Tables [file 41408_2024_983_MOESM1_ESM.pdf]
